# Photoresist-Free Patterning by Mechanical Abrasion of Water-Soluble Lift-Off Resists and Bare Substrates: Toward Green Fabrication of Transparent Electrodes

**DOI:** 10.1371/journal.pone.0083939

**Published:** 2013-12-17

**Authors:** Adam D. Printz, Esther Chan, Celine Liong, René S. Martinez, Darren J. Lipomi

**Affiliations:** Department of NanoEngineering, University of California San Diego, San Diego, California, United States of America; University of South Carolina, United States of America

## Abstract

This paper describes the fabrication of transparent electrodes based on grids of copper microwires using a non-photolithographic process. The process—“abrasion lithography”—takes two forms. In the first implementation (Method I), a water-soluble commodity polymer film is abraded with a sharp tool, coated with a conductive film, and developed by immersion in water. Water dissolves the polymer film and lifts off the conductive film in the unabraded areas. In the second implementation (Method II), the substrate is abraded directly by scratching with a sharp tool (*i.e.*, no polymer film necessary). The abraded regions of the substrate are recessed and roughened. Following deposition of a conductive film, the lower profile and roughened topography in the abraded regions prevents mechanical exfoliation of the conductive film using adhesive tape, and thus the conductive film remains only where the substrate is scratched. As an application, conductive grids exhibit average sheet resistances of 17 Ω sq^–1^ and transparencies of 86% are fabricated and used as the anode in organic photovoltaic cells in concert with the conductive polymer, poly(3,4-ethylenedioxythiophene):poly(styrenesulfonate) (PEDOT:PSS). Compared to devices in which PEDOT:PSS alone serves as an anode, devices comprising grids of copper/nickel microwires and PEDOT:PSS exhibit lowered series resistance, which manifests in greater fill factor and power conversion efficiency. This simple method of forming micropatterns could find use in applications where cost and environmental impact should be minimized, especially as a potential replacement for the transparent electrode indium tin oxide (ITO) in thin-film electronics over large areas (i.e., solar cells) or as a method of rapid prototyping for laboratory-scale devices.

## Introduction

Transparent electrodes are essential components of nearly all displays, touch screens, and thin-film photovoltaic devices. The global market for transparent electrodes is expected to grow from $2 billion in 2012[1] to at least $10 billion by 2016.[2] Thin-film solar installations, in particular, will likely expand the market for transparent electrodes even further in the near future. Indium tin oxide (ITO) comprises ninety percent of the global market. Indium, however, has an abundance in the Earth's crust of 0.05 ppm, and its cost has risen an order of magnitude over the past decade.[3] The imminent scarcity of indium has fueled an enormous amount of research on potential replacements for ITO for large-area and laboratory-scale optoelectronic devices. Prominent examples of next-generation materials include carbon nanotubes[4], [5] and graphene,[6] silver nanowires,[7], [8] and printed metallic grids produced by high-resolution photolithography or nanoimprint lithography.[9] Many of these materials suffer from high energies of production, use of proprietary materials, or natural abundances as low as that of indium (*e.g.*, silver). A transparent electrode can only comprise a fraction of the cost of a thin-film photovoltaic module, which, according to the analysis by Lewis and Nocera, may only be $10 m^–2^ in total to compete with fossil fuels for primary energy.[10] Any method of producing transparent electrodes for thin-film photovoltaic devices must be manufacturable on the scale of thousands of square kilometers for approximately the cost of paint.[11]

**Figure 1:**
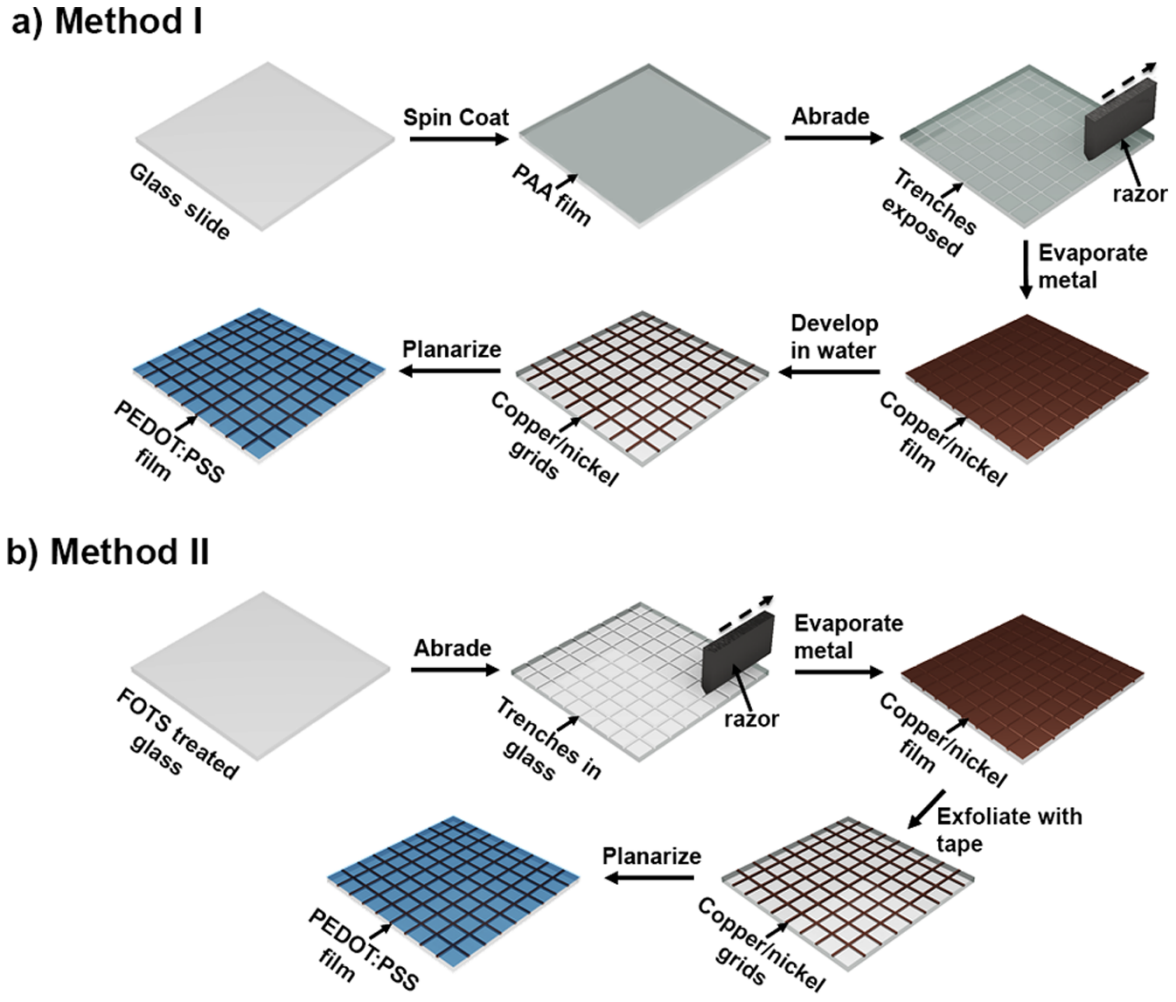
Schematic diagrams summarizing the two implementations of abrasion lithography. (a) Method I uses mechanical abrasion with a sharp tool to pattern water-soluble thin films. (b) Method II produces patterns by direct abrasion of glass substrates.

Our goal was to generate functional micropatterns for transparent electrodes using an extremely simple method and the greenest, least expensive materials possible. We describe an approach to micropatterning at whose core is mechanical abrasion of surfaces followed by lift-off in the unabraded regions. We have nicknamed the process “abrasion lithography.” Abrasion lithography can be used in one of two ways. In the first method (Method I, **Figure 1a**), a sharp tool was used to scratch furrows in a water-soluble commodity polymer, which behaved as a lift-off resist. Following blanket deposition of a thin conductive film, the substrate was immersed in water, the commodity polymer dissolved, and the conductive film remained on the surface only in the abraded regions. In the second method (Method II, **Figure 1b**), the substrate was abraded directly, with no lift-off resist needed. Blanket deposition of a thin conductive film left material in the recessed furrows created by the abrasion. Lift-off of the unabraded areas with adhesive tape again created conductive patterns defined by the mechanical patterning.

### Background

#### Transparent electrodes

Transparent electrodes fall roughly into two categories, those comprising contiguous films of conductive materials that are partially transmissive to optical wavelengths when sufficiently thin, and those comprising percolated networks of high-aspect-ratio conductive particles. Prominent examples of contiguous films exhibiting high transparency include tin-doped indium oxide (ITO), the conductive polymer poly(3,4-ethylenedioxythiophene):poly(styrene sulfonate) (PEDOT:PSS),[12]–[15] ultrathin films of gold,[16] and single- or few-layer graphene.[6], [17], [18] Examples of percolated networks include films of carbon nanotubes,[19]–[21] solution-grown silver[22] and copper nanowires,[3], [23], [24] nanowires based on electrospun templates,[25], [26] patterns created by the coffee ring effect,[27] and various implementations of microcontact printing[28], [29] and nanoimprint lithography.[30], [31] Deposition of reduced graphene oxide sheets onto a surface can also form percolated networks;[32] these films occupy a middle ground between the contiguous and percolated categories. Transparent electrodes exhibiting either a contiguous or percolated structure are not directly interchangeable in a given application. For example, while electrodes comprising percolated networks of particles may be used in touch screens and certain types of displays, they generally need to be combined with films of the contiguous variety in order to inject or extract charge from devices in which the semiconductor has low mobility—*i.e.*, organic light-emitting devices and solar cells.[33]

“Energy cannibalism” refers to the inputs of energy required to generate additional energy.[34] An implicit goal of all research in renewable energy technologies should be to reduce the cumulative energy demand of the product during its manufacture.[35] While ultra-thin organic solar cells have the potential to be low-cost, low-energy solutions to the growing global demand for energy—in fact, they have the highest ratio of power-to-mass of any thin-film photovoltaic technology,[36] 10 W g^–1^—the production energy of such a device is finite, and should be minimized. According to the analysis by Anctil *et al.*, the largest single contributor to the cumulative energy demand of an organic solar cell is the transparent electrode.[35] Sputter-deposition of ITO on polyethylene terephthalate (PET) substrates represents 39%–50% of the production energy of polymer-fullerene and small-molecule-based solar cells.[35] The contribution of printing silver contacts adds an additional 8%–11% to the cumulative energy demand, while the contribution of PEDOT:PSS (present in essentially all organic solar cells) is negligible.[35] While the correlation is not perfect, production energy tends to scale with cost, and thus reducing the energy demand of the transparent electrodes and printed contacts should help meet the “double bottom line” possible with organic solar cells.[37] That is, to generate energy inexpensively using materials and processes that do not degrade the environment by their method of extraction or their production energy manifested in increased emissions of carbon dioxide. The goal of this research is to evaluate a potentially green and inexpensive alternative to depositing and patterning conductive electrodes based on indium, silver, specialty chemicals such as photoresist, and processing using organic solvents.

## Experimental Design

### Choice of copper

We chose to fabricate transparent electrodes based on copper wires because of its price and conductivity. After silver, copper is the second-most conductive element. At a price of roughly $7 kg^–1^,[38] it is two orders of magnitude less expensive than silver or indium, and has been so since at least 1970.[39] Additionally, its abundance in the Earth's crust is 1000 times greater than that of either silver or indium,[40] which suggests the lower price of copper will remain intact for the foreseeable future. While copper micro- and nanostructures degrade in the ambient over time by oxidation, this characteristic is shared by silver.[24] By cladding copper structures with nickel, they can be protected from oxidation.[24]

### Poly(acrylic acid) as water-soluble lift-off resist

We chose poly(acrylic acid) (PAA) for its ability to form thin films when coated from an aqueous solution, its brittleness (that is, its amenability to patterning by mechanical abrasion) and its ability to be crosslinked by divalent metal cations in anticipation of the need to process it orthogonally to other water-soluble materials.[41], [42] PAA has the advantage of being a commodity polymer with an extraordinarily low cost (∼$1 kg^–1^),[43] which is several orders of magnitude less expensive than photoresist based on poly(methyl methacrylate) (PMMA), diazonapthoquinone-novolac (DNQ-novolac), or epoxy (*e.g.*, SU-8). Its developer, water, is the least expensive, most environmentally benign solvent available, and thus compares favorably to the solvents used to process the above photoresists: acetone, aqueous tetramethyl ammonium hydroxide (TMAH), and propylene glycol methyl ether acetate (PGMEA). PAA readily dissolves in non-potable water used in industrial processes, thus deionized water is not required.

### Metallic grids as transparent electrodes

We believe that grids of metallic wires in a deterministic pattern have some advantages (as well as disadvantages) when compared to randomly deposited nanowires grown from solution. Metallic grids created by a single step have no potentially resistive junctions between wires and thus no requirement to weld them together to improve conductivity.[44] Grids, moreover, are automatically percolated and do not contain isolated structures or widowed termini that do not carry current but detract from the optical transmission. The theoretical conductivity and transparency is also easily calculated from the geometry.[45] The shortcomings of grids derived from an evaporated film are that they are not easily amenable to processing from solution and that deposition under vacuum is energy intensive. Vacuum metallization does not preclude large-scale production, however. For example, biaxially oriented polyethylene terephthalate (BoPET) is metallized by physical vapor deposition and is a ubiquitous, low-cost material for food packaging (e.g., potato chip bags), and can be obtained for (<$1 m^–2^).[46] Roll-to-roll evaporative processing and liftoff in the context of electrodes has been demonstrated with flexographic printing, however, the features had a much lower resolution (100 µm) than is attainable with abrasion lithography.[47] It may also be possible to develop solution-based methods of blanket deposition of conductive particles that land in the regions patterned by abrasion and that do not detach upon dissolution of the PAA in water.[48]

### Mechanical processing

The core of the project is the use of mechanical abrasion—*i.e.*, machining—to generate patterns inexpensively. Mechanical processing by scratching with a sharp tool could be replaced by stretching, intentional cracking, or other mechanical force intended to produce percolated networks of furrows.[49]–[51] We tested sharp metallic (steel razor blades) and relatively softer polymeric (polypropylene picnic knives) tools for abrasion. The advantage of steel razor blades was their narrow edge. The advantage of the polypropylene knives was that there was a reduced tendency to penetrate through the PAA film and scratch the substrate. We chose these tools on the basis of cost and we are certain that purpose-fabricated cutting tools would produce a significantly higher pitch and resolution than we were able to achieve with the simple, commercial tools we chose.

### Application in organic solar cells

As an application to evaluate the utility of mechanical abrasion as a lithographic tool, we fabricated copper grids as transparent electrodes for organic solar cells. We chose to use devices based on a bulk heterojunction of poly(3-hexylthiophene):[6,6]-phenyl C_61_ butyric acid methyl ester (P3HT:PCBM) due to its prominence in the literature.[52] We used PEDOT:PSS as a hole-transporting layer and eutectic gallium-indium as a low-work-function top electrode.[53] We found that PEDOT:PSS, when spin-coated directly on top of the grids, degraded the copper, and thus found it necessary to evaporate a layer of nickel to protect the copper from damage. This concept was previously demonstrated by Rathmell *et al.* in a one-pot synthesis of cupronickel nanowires, which exhibited resistance to oxidation in ambient air.[24]

## Materials and Methods

### Materials

Glass substrates were 7.5 mm×5.0 mm×1.0 mm microscope slides obtained from Premiere. Poly(acrylic acid) (PAA) was purchased as a 25 wt% solution in water from Alfa Aesar. PEDOT:PSS (Clevios PH1000) was purchased from Heraeus. The solid content of the PH 1000 solution was 1–1.3% and had a ratio of PEDOT to PSS of 1∶2.5 by weight. (Tridecafluoro-1,1,2,2,-tetrahydrooctyl)-trichlorosilane (FOTS) was purchased from Gelest. Zonyl FS-300 (Zonyl), dimethyl sulfoxide (DMSO), ortho-dichlorobenzene (ODCB), poly(3-hexylthiophene) (P3HT), [6,6]-phenyl C_61_ butyric acid methyl ester (PCBM, >99%), and eutectic gallium-indium (EGaIn, ≥99.99%) were purchased from Sigma-Aldrich and used as received. All other solvents were purchased from Fisher Scientific or VWR International and used as received. Leitsilber 200 silver paint was purchased from Ted Pella and used as received.

### Preparation of substrates

Glass slides were cleaned with Alconox solution (2 mg mL^–1^), deionized water, acetone, and then isopropyl alcohol (IPA) in an ultrasonic bath for 10 min each and then rinsed and dried with compressed air. Next, the glass was plasma treated at ∼30 W for 3 min at a base pressure of 200 mtorr ambient air to remove residual organic material and activate the surface. For samples patterned by Method I, a solution of 6 wt% PAA in 18 wt% water and 76 wt% IPA was spin coated onto the glass slides at a speed of 2000 rpm (500 rpm s^–1^ ramp) for 60 s. These conditions produced a film of PAA with a thickness of ∼400 nm (as measured by stylus profilometry). For samples patterned by Method II, the slides were placed in a vacuum desiccator with a glass vial containing ∼100 µL of FOTS and put under house vacuum for a minimum of 3 h to passivate the surface.

### Scoring process

We then scored the samples with a steel razor (Method I and II) or polypropylene knife (Method I only). Scoring was done by hand using a purpose-built linear motorized stage with an attached acrylic straight edge (which was used like a draftsman's T-square). The limit of the pitch is determined by the resolution of the linear motorized stage. For our experiments the apparatus permitted scratching of parallel grid lines in increments ≥100 µm. A similar apparatus which has a resolution of 500 µm could be built for ∼$500, which is reasonable for laboratory-scale rapid prototyping. For this set of experiments, the film was scored with a pitch of 500 µm with the razors and 2000 µm with the polypropylene knives. While scoring with the razor in Method I, we found it difficult to prevent the razor from scratching the glass substrate beneath the water-soluble film. After scoring the entire surface in one direction, the glass was rotated 90° and then scored again to create a grid of orthogonal lines. A new scoring tool (steel razor or polypropylene knife) was used for each sample to improve repeatability of the procedure. After scoring, excess debris was blown from the substrate using a stream of compressed air. To estimate the pressure applied while scoring, we secured a sample to a balance and scored the samples with a razor or polypropylene knife. We obtained the pressure by dividing the force measured by the balance by the area of the cutting tool in contact with the sample as measured by SEM.

### Deposition of films

After scoring the water-soluble resist (Method I) or the glass substrate (Method II), we evaporated copper (50 nm) and nickel (10 nm) onto the surface by electron-beam evaporation. For the samples produced by Method I, the microwire grids were developed by immersion in water. To increase the diffusion of water into the water-soluble layer, ultrasonication in a water bath for at least 10 min was generally required. The remaining water-soluble layer and excess metallic film was subsequently removed by rinsing with water. For the samples produced by Method II, the microwire grids were developed by exfoliation with Scotch tape. To ensure the tape contacted the entire film surface, gentle pressure was applied with a pair of tweezers.

### Optical transparency of copper/nickel microwire grids

The optical transparency (%T) was measured using a PerkinElmer Lambda 1050 UV-vis-NIR spectrophotometer. The wavelength range measured was 850-350 nm with a step size of 5 nm. Blank glass slides were cleaned and plasma treated under the same conditions as slides for sample preparation.

### Sheet resistance (*R_s_*) measurements

We measured electrical resistance by isolating rectangular strips (*w* = 0.5 cm, *l*∼1–2 cm) from the microwire grids using a diamond scribe. We painted silver contacts on the termini of each rectangular strip. The resistance of the electrodes was measured using a Keithley 2400 SourceMeter. We measured the distance from the leading edge to leading edge of the silver paint electrical contacts with a caliper and calculated the *R_s_* according to the relationship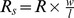, where *w* and *l* were the widths and lengths of the rectangular regions of the microwire grids.

### Imaging

Optical micrographs were taken using a Zeiss Axio Fluorescence Microscope. The widths of the copper/nickel microwires were calculated using the measurement tool in Adobe Photoshop. Due to the line-edge roughness, each wire was measured at seven different points (selected arbitrarily). Atomic Force Microscopy (AFM) micrographs were taken using a Veeco Scanning Probe Microscope in tapping mode. Data was analyzed with NanoScope Analysis v1.40 software (Bruker Corp.). The samples observed in AFM were 1 cm^2^ cut by diamond-tipped scribe from larger glass slides after the scoring process. To remove glass debris from the surfaces, the samples for AFM were ultrasonicated in IPA for 10 min and subsequently rinsed with additional IPA and dried using compressed air. Scanning electron microscope (SEM) micrographs of the scoring tools were performed using a FEI XL30-SFEG SEM at 5 kV.

### Fabrication of organic solar cells

We placed the developed grids in a glass container filled with IPA and then placed the containers in a bath ultrasonicator for 20 min to remove accumulated debris. The samples were then rinsed with IPA and dried with compressed air. We subsequently deposited the PEDOT:PSS layer for both the electrodes with and without microwire grids from an aqueous solution containing 92.1 wt% Clevios PH 1000 (∼0.9–1.2 wt% PEDOT:PSS), 6.9 wt% DMSO, and 1 wt% Zonyl. The concentration of DMSO was reported to be optimal for solar cell fabrication,[54] and the zonyl was added to increase the conductivity of the PEDOT:PSS.[13] The solution was filtered with a 1 µm glass microfiber (GMF) syringe filter and then spin coated at a speed of 500 rpm (100 rpm s^–1^ ramp) for 60 s, followed by 2000 rpm (750 rpm s^–1^ ramp) for 60 s, which resulted in a layer 200 nm thick. The samples were subsequently dried at 150°C for 30 minutes under a Pyrex petri dish covered in aluminum foil to reduce dissipation of heat and to prevent dust from landing on the samples. After 30 min of drying, the samples were left to cool to room temperature for 30 minutes while still covered on the hotplate.

The photoactive layer was deposited from a solution of 1∶1 by weight P3HT and PCBM in ODCB (40 mg mL^–1^), which was stirred overnight and filtered in a 0.20 µm poly(tetrafluoroethylene) (PTFE) syringe filter. The solution was then spin coated onto the electrode layer at a speed of 500 rpm (100 rpm s^–1^ ramp) for 240 s, followed by 2000 rpm (750 rpm s^–1^ ramp) for 60 s; these conditions produced a layer 230 nm thick. A thin strip of the microwire grid/PEDOT:PSS electrode was exposed by wiping away some of the P3HT:PCBM film with chloroform so that electrical contact could be made. The samples were then immediately placed in a nitrogen-filled glovebox and annealed at 125°C for 30 min under a Pyrex petri dish covered in aluminum foil. The substrates were then allowed to cool slowly to room temperature. To minimize exposure to ambient air by transferring devices into and out of an evaporator in a different building, EGaIn (extruded by hand from a syringe) was used as the top contact.[55], [56]

### Photovoltaic characterization of organic solar cells

The photovoltaic properties were measured in a nitrogen-filled glovebox using a solar simulator with a 100 mW cm^–2^ flux that approximated the solar spectrum under AM 1.5G conditions (ABET Technologies 11016-U up-facing unit calibrated with a reference cell with a KG5 filter). The current density versus voltage was measured for both dark and under illumination using a Keithley 2400 SourceMeter.

## Results and Discussion

### Grids produced by abrasion of water-soluble lift-off resist (Method I)

**Figure 2:**
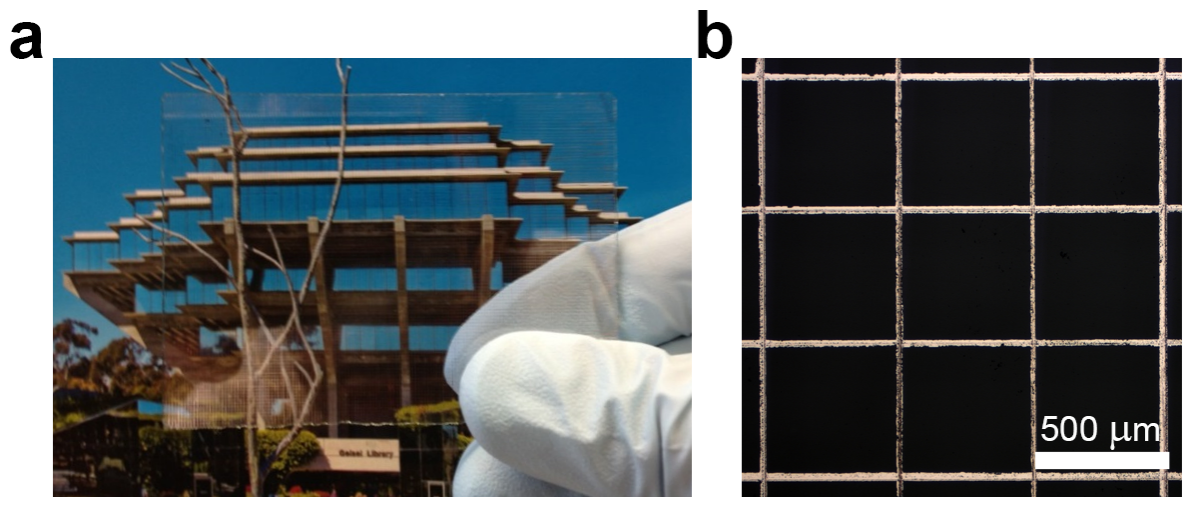
Images of transparent electrodes fabricated by abrasion lithography. (a) Photograph showing the high transparency of grids produced by Method II. A reflection in the bottom left corner of the glass substrate shows the copper wires. (b) Optical micrograph showing the wires produced by Method II.

**Figure 3:**
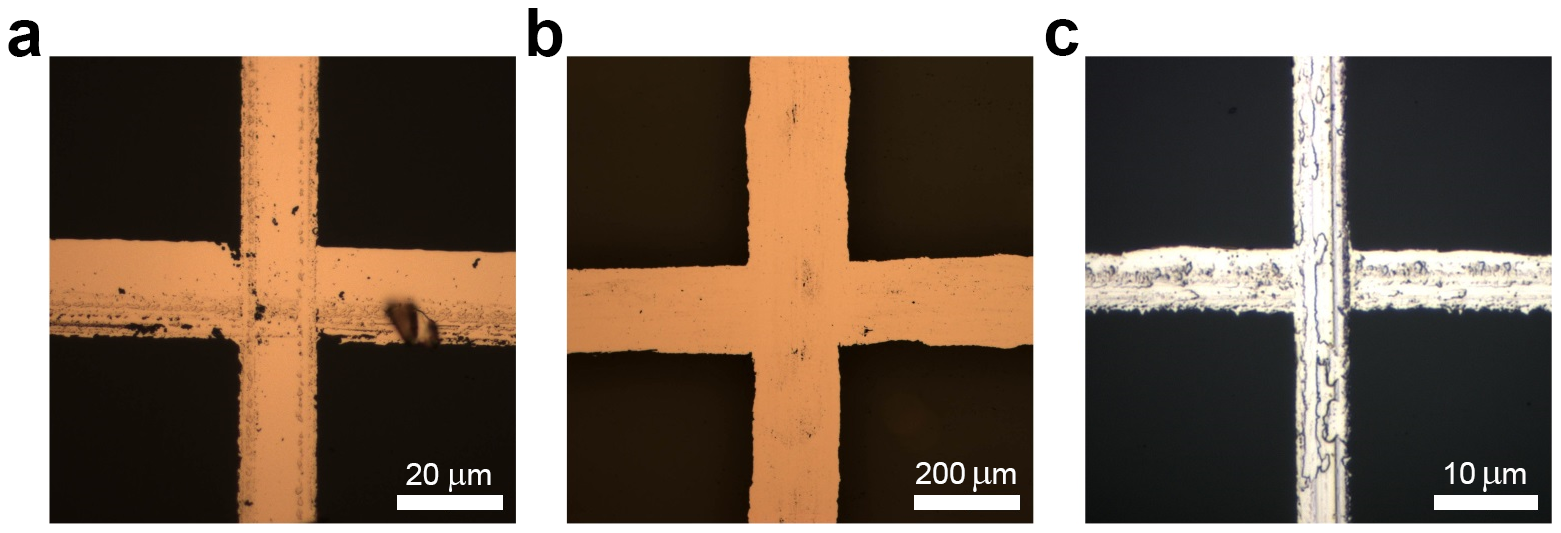
Optical micrographs of junctions of copper and copper/nickel microwires. (a) A microwire junction fabricated by Method I. Surface roughness caused by the razor inadvertently abrading the substrate is apparent. (b) A microwire junction fabricated by patterned a PAA film with a polypropylene picnic knife, which was too soft to abrade the glass substrate, and thus the microwires appear to have a smoother topography. (c) A microwire junction patterned by direct abrasion of glass by a steel razor. Significant roughness generated by the razor is clearly visible.

**Figure 4:**
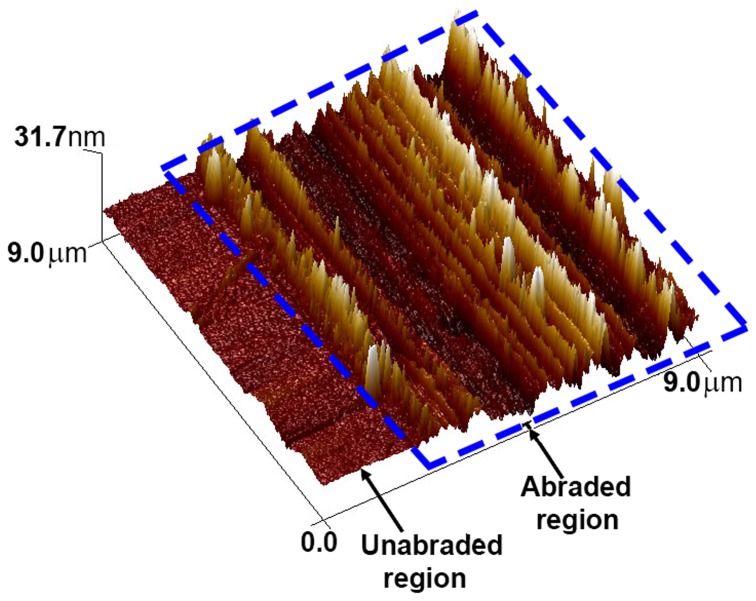
3D AFM image of glass after scoring using a steel razor. The abraded region has been roughened by the steel razor blade, while the unabraded region is relatively flat. The abraded region has been highlighted by the dashed blue line.

We successfully generated grids using both Method I and Method II. **Figure 2a** shows a photograph that demonstrates the transparency of grids produced by Method II. The transparency is due to the thinness of the microwires relative to their pitch as shown in **Figure 2b** **.** **Figure 3a** shows a junction of copper microwires produced by scoring PAA films on glass substrates with steel razor blades. The minimum linewidth we were able to achieve was 11 µm (average linewidth was 29±10 µm, *N* = 839, as determined by measurement of optical micrographs in Adobe Photoshop) using a razor blade with an initial edge width of less than 1 µm. From the optical micrograph in **Figure 3a**, it is apparent that the steel razor blades also abraded the surface of the glass beneath the PAA film. We confirmed this observation by AFM (**Figure 4**). The abrasion in the glass surface by the steel razor blades appeared to introduce substantial roughness in the abraded regions. When measured by AFM, we found the abraded regions to have a root mean square roughness of ∼10 nm compared to ∼1 nm in the unabraded regions. The roughness in the abraded regions translates to the wires in the electrodes because evaporative metal deposition is a vertically conformal process. However, this surface roughness is much smaller than the height of the wires, so it is unlikely to be significantly detrimental to performance. (We exploited this roughening of the glass with direct abrasion of the glass substrates in Method II.) We also explored polymeric cutting tools. We tested the performance of a polypropylene picnic knife whose edge had a radius of curvature of approximately 40 µm, as determined by scanning electron microscopy. **Figure 3b** shows a junction of copper wires produced using this cutting tool, which have a minimum linewidth of 246 µm (the average linewidth was 346±45 µm, N = 840). The amount of pressure with the razor was estimated to be 2–11 GPa after the first score (“cycle”); the maximum pressure of the first score was estimated to be on the order of 150 GPa assuming that the blade edge was the width of a new blade; because the edge of the blade dulled with each cycle, however, this estimate was likely high (dulling is discussed below). The pressure applied by the polypropylene knife was estimated to be 80–140 kPa, which we calculated with the assumption that only one of the serrated teeth was in contact with the substrate at any given time.

### Grids produced by direct abrasion of the substrates (Method II)

During the course of developing Method I, we made the serendipitous discovery that the water-soluble lift-off resist was not necessary to produce conductive grids, and that the copper microwires patterned by direct abrasion of the substrate (Method II) could be of thinner linewidth than those produced by Method I. **Figure 3c** shows a junction produced by direct abrasion of a glass substrate using a steel razor blade, blanket deposition of copper/nickel, and lift-off from the unabraded areas using Scotch tape. The minimum linewidth achieved was less than 5 µm (average linewidth was 17±4 µm, N = 70). While the mechanism of Method I is analogous to a lift-off process in conventional lithography, the primary mechanism of Method II appears to be based, in order of importance, on three effects. The first effect is the inability of the adhesive tape to penetrate into the recessions in the substrate created by the razor blade. This supposition is supported by the observation that thicker copper films (>100 nm) lift off in the abraded regions. The second effect is the increased roughness of the abraded regions, which increases the van der Waals force per unit area between the glass and the copper. The third effect is the scratching away of the fluorinated alkyl silane, which was used to passivate the glass in the unabraded regions to facilitate lift-off with tape.

**Figure 5:**
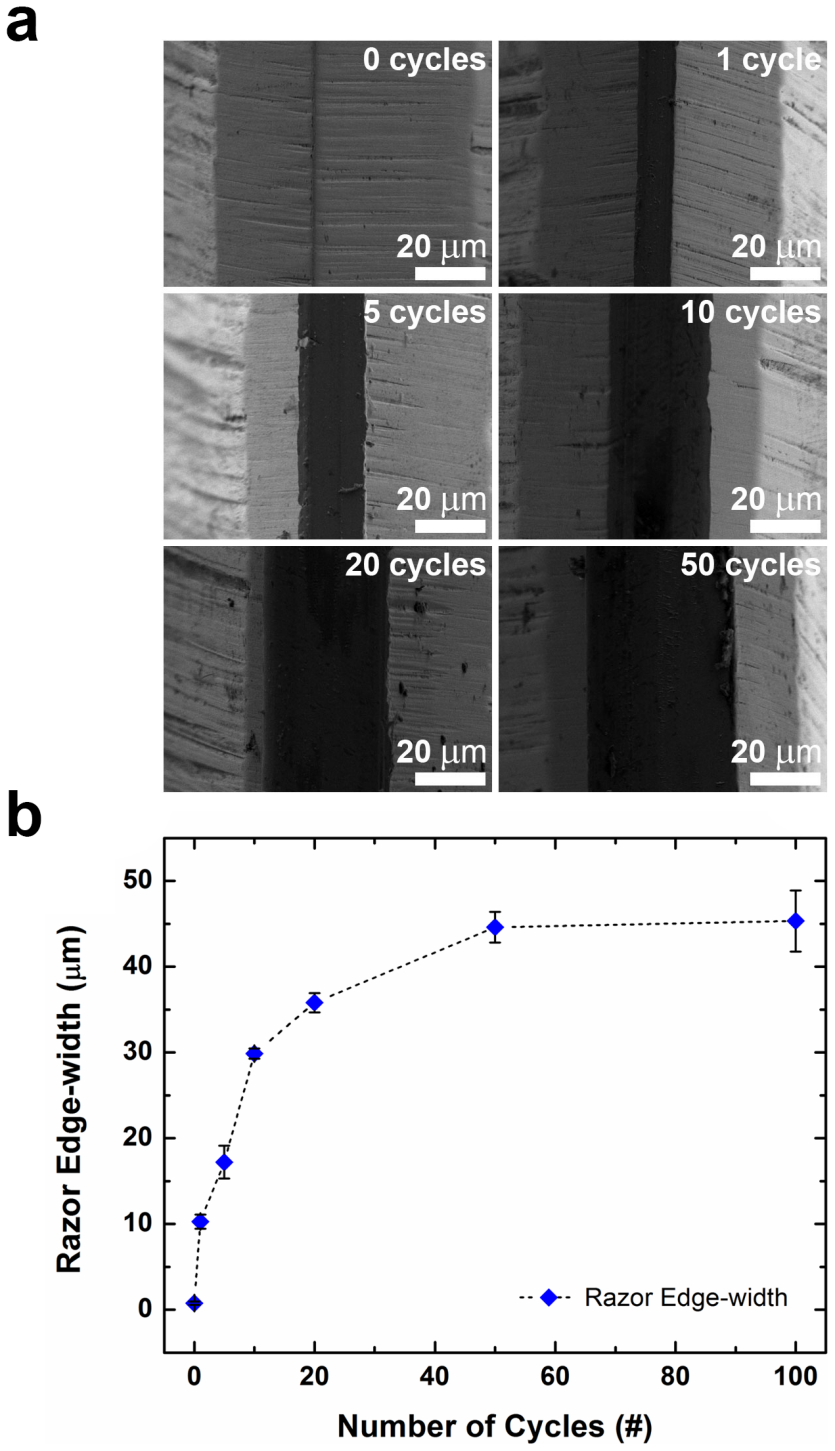
Dulling of cutting tools. (a) SEM micrographs showing the progression of the dulling of a razor used in Method II, from out of box to 50 cycles. (b) The dulling of the razor plateaus at around 50 cycles.

We observed dulling of the razor blades when used on a glass substrate in both Methods I and II. After the first cycle, the razor dulled from an edge width of less than 1 µm to 10 µm; after 100 cycles, the edge width was approximately 45 µm (**Figures 5a, b**). We believe applying lower pressure using an automated apparatus or using a cutting tool made of a harder material could minimize dulling. Purpose-made cutting tools composed of harder materials such as tungsten carbide or diamond would likely degrade at a much slower rate than the steel razors we evaluated.

### Performance of copper grids as transparent electrodes

**Table 1** summarizes the pitch, linewidth, and experimental and theoretical sheet resistance, transparency, and figure-of-merit of the grids of the types depicted in **Figure 3**. We calculated the theoretical transparency according to equation 1, 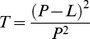
(1)where *T* is the transparency of the sample, *P* is the pitch of the microwire grid, and *L* is the average linewidth of the microwires. The theoretical sheet resistance was calculated according to the method used by Catrysse and Fan,[45]

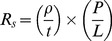
(2)where *ρ* and *t* are the resistivity and the thickness of the copper, respectively. We took the resistivity to be that of bulk copper, 16.8×10^–9^ Ω m. The figure-of-merit (FOM) was calculated according to equation 3, 
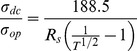
(3)


**Table 1:** Summary of the properties of the electrodes of the types depicted in Figure 3.

Electrode Type		Pitch (µm)	Linewidth (µm)	Expl. Sheet Resistance (Ω sq^–1^)	Calc. Sheet Resistance (Ω sq^–1^)	Expl. Transparency (%)	Calc. Transparency (%)	Expl. Figure of Merit	Calc. Figure of Merit
PEDOT:PSS		–	–	86±7	–	88.0±0.3	–	33	–
Water-soluble resist, plastic knife patterned	w/o PEDOT:PSS	2000	346±45	5.7±1.6	1.93	68.7±4.4	68.4	160	465
Water-soluble resist, plastic knife patterned	w/PEDOT:PSS	2000	346±45	9.9±5.2	–	63.9±5.2	–	76	–
Water-soluble resist, razor patterned	w/o PEDOT:PSS	500	29±10	17±7	5.76	86.2±3.9	88.7	140	530
Water-soluble resist, razor patterned	w/PEDOT:PSS	500	29±10	68±63	–	78.0±3.7	–	21	–
Direct abrasion w/razor	w/o PEDOT:PSS	500	17±4	59±40	9.8	88.5±3.0	93.3	50	544

*R*_s_. Figures of merit were calculated from the average transparency and

Where *σ_dc_* is the dc conductivity and *σ_op_* is the optical conductivity. Based on the FOM, the best devices were obtained by Method I using the polypropylene knife to pattern the films.

Deviations of the experimental sheet resistance from the theoretical sheet resistance were caused predominantly by line-edge roughness for the samples prepared by Method I and poor junctions for the samples prepared by Method II. Because these samples were made by hand, there was variability introduced by inconsistently applied pressure from cycle to cycle. The effects of these inconsistencies include breaks in individual wires and poor junctions, as well as significant line-edge roughness. An additional explanation for breaks in the wires and poor junctions for samples prepared by Method I is a failure to completely clear the furrows of water-soluble resist. This was likely an effect of the vibrations introduced by manual scoring. For Method II, the inconsistent pressure applied by manual scoring caused variations in the depths of furrows. In some areas, the furrows were not large enough to protect the metal from exfoliation by the adhesive tape. We believe that automation of these processes would resolve these problems, in part, and thus we believe the FOM could be substantially improved as well. The calculated FOMs based on the geometry of our grids in **Table 1** suggest that improvement should be possible.

It is worth noting the increase in *R_s_* with the addition of the PEDOT:PSS planarizing layer. This observation suggests that oxidation of the copper microwires, most likely by the sulfonic acid present in PEDOT:PSS, lowered the conductivity of the grids. This oxidation likely occurs through gaps in the microwire surface that are left unprotected by the nickel due to the surface roughness and directional nature of evaporative deposition. We believe that this problem could be resolved by a conformal deposition process, such as sputter-coating or electroless plating with nickel.[24] (It should be noted that while a planarizing layer such as PEDOT:PSS is necessary to facilitate efficient charge collection for organic photovoltaics, it is not necessary in other applications for transparent electrodes such as touchscreens.) We calculated FOM values of metallic grid electrodes with PEDOT:PSS assuming that there was no oxidation of the electrodes.

The FOMs of grids produced by Method I were 160 and 140 for the water-soluble resist patterned by polypropylene knife and steel razor, respectively. This FOM was lower than, but still of the same order of magnitude of commercially available ITO, which had been reported to have a FOM between 400–800.[57] The theoretical FOMs in **Table 1** suggest that if defects are minimized, the grids with the current geometry should be able to perform similarly to ITO, with FOMs of 465 (patterned by the polypropylene knife) and 530 (patterned by the steel razor) at a greatly reduced cost. With PEDOT:PSS added, due to a reduction in transparency, these FOMs drop slightly to 337 and 248 for the polypropylene knife patterned and the steel razor patterned, respectively. These values compared favorably with copper/nickel nanowire networks (transparency of 84%, *R_s_* of 60 Ω sq^–1^, and FOM ∼34),[24] carbon nanotube networks (transparency of 90.1%, *R_s_* of 60 Ω sq^–1^, and FOM ∼59),[20] and graphene (transparency of 90%, *R_s_* of 30 Ω sq^–1^, and FOM ∼116).[58] Silver nanowire networks (transparency of 75%, *R_s_* of 3.4 Ω sq^–1^, and FOM ∼357),[57] copper networks fabricated by deposition onto electrospun polymer (transparency of 90%, *R_s_* of 2 Ω sq^–1^, and FOM ∼1738),[26] and metallic (gold) grids fabricated by photolithography (theoretical reported transparency of 90%, *R_s_* of 0.8 Ω sq^–1^, and FOM ∼4344)[45] either performed as well or better than the electrodes reported here, but there are significant disadvantages to each of these fabrication methods. The FOM of transparent electrodes comprising of silver nanowire networks is partially limited by junction resistance.[44] Conversely, the electrodes produced by our methods can have contiguous junctions. Electrospinning is currently less scalable than the abrasion lithography methods, and abrasion lithography may have advantages over photolithography for laboratory-scale fabrication and possibly for large-area applications.

### Performance of grids in organic solar cells

**Figure 6:**
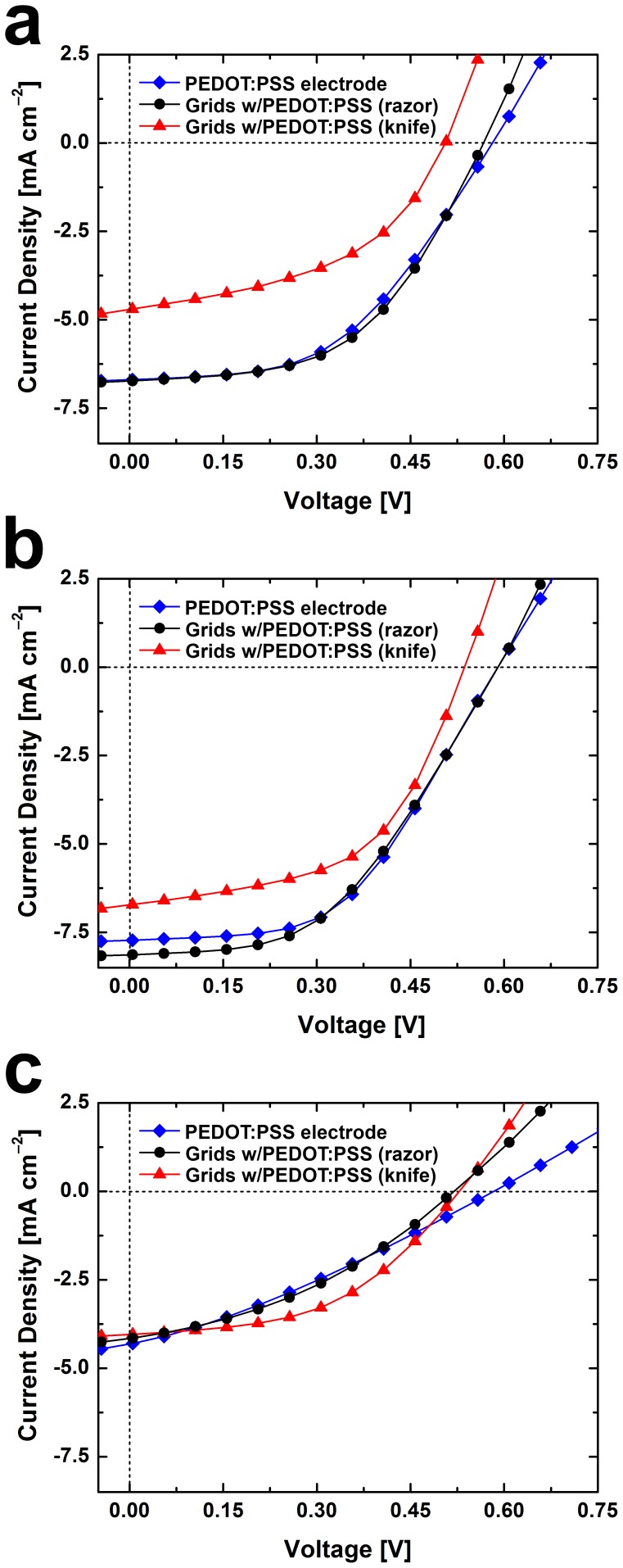
*J-V* curves of P3HT:PCBM solar devices. (a) The average *J-V* curves for devices with transparent electrodes consisting of PEDOT:PSS (*N* = 7), PEDOT:PSS and grids patterned by polypropylene knife (*N* = 6), and PEDOT:PSS and grids patterned by steel razor (*N* = 4). (b) The *J-V* curves for the highest efficiency cells from the sample sets from (a). (c) The *J-V* curves for the highest efficiency larger cells (∼0.5 cm^2^ compared to ∼0.1 cm^2^ in (a) and (b)).

To evaluate the potential of grids produced by abrasion lithography to improve the performance of organic photovoltaic devices, we fabricated devices by depositing grids consisting of copper (50 nm) and nickel (10 nm) produced by Method I, PEDOT:PSS, P3HT:PCBM, and a top electrode of eutectic gallium indium (EGaIn). **Figure 6a** shows plots of the average current density vs. voltage (*J-V*) of devices with and without grids (“small” device areas, ∼0.1 cm^2^). The control samples (PEDOT:PSS only) and the samples with grids patterned by steel razor performed similarly. The average short circuit current (*J_sc_*) for the control cells was 6.7±1.4 mA cm^–2^ compared to 6.7±1.3 mA cm^–2^ for cells with grids patterned by steel razor and 5.2±1.3 mA cm^–2^ for cells with grids patterned by polypropylene knife. Additionally, the average open-circuit voltage (*V_oc_*) for the control cells was 580±14 mV compared to 570±20 mV for the cells with grids patterned by steel razor and only 510±40 mV for the cells with grids patterned by polypropylene knife. While the samples with grids patterned by steel razor performed with the same efficiency as the control samples (1.9±0.3% for the controls compared to 2.0±0.3% for the cells with grids patterned by steel razor), the samples with grids patterned by polypropylene knives were about 0.6–0.7% less efficient. This effect is due to the reduced *J_sc_* which we attribute to the lower transparency when compared to that of the control devices. The *J-V* curves of the cells with the highest measured efficiency (*η_e_*) from the sample sets in **Figure 6a** are plotted in **Figure 6b**. These “hero” cells exhibit similar trends as the average values. Regardless of the patterning tool, the average device with grids had a reduced series resistance (*R_series_*) when compared to the control devices. The average control cell had an *R_series_* of 30±6 Ω cm^2^ compared to 20±5 Ω cm^2^ and 16±9 Ω cm^2^ for cells with grids patterned by steel razor and polypropylene knife, respectively. The reduction in *R_series_* is directly attributable to the higher conductivity of copper when compared with PEDOT:PSS alone. We reasoned that with larger cells, the effect of lowered *R_series_* of the devices with grids might allow them to outperform the devices without grids. To demonstrate this effect, we increased the size of the cells from ∼0.1 cm^2^ to ∼0.5 cm^2^. **Figure 6c** shows the plots of the *J-V* curves of the larger cells with the highest *η_e_*. This plot highlights the effect of the grids for large-area cells, namely reduced *R*_series_, and increased fill factor (*FF*). We attribute the increase in *FF*, in part, to the reduced *R_s_* of the transparent electrodes that contained grids along with PEDOT:PSS when compared to the PEDOT:PSS electrodes without grids. For these large-area cells, the *FF* of the representative PEDOT:PSS device dropped to 30% from 50% when compared with the representative smaller-area device, while the devices with the grids patterned by steel razor and by polypropylene knife only dropped to 37% from 47% and 48% from 53%, respectively. The most efficient of the larger devices contained grids patterned by plastic picnic knives because the overall reduction in sheet resistance overcame the reduced *J_sc_* due to attenuated absorption.

## Conclusions

This paper described a simple method of fabricating grids of microwires using green, inexpensive materials. Like the use of biaxially pre-strained polymeric sheets (*i.e.*, the “shrinky-dink” method) for fabricating topographic masters for microfluidics,[59], [60] the use of transparency masks for photolithography,[61] and the use of wax printing for paper-based diagnostics,[62] we believe that abrasion lithography could be a useful tool for low-cost and environmentally benign micro—and possibly nano—fabrication for rapid prototyping and generating simple patterns over large areas. The minimal tools required are methods of depositing polymeric and conductive films, and the minimal materials required are poly(acrylic acid) (the absorbent found in diapers and artificial snow) and steel razor blades (or polypropylene picnic knives). The ability to pattern by direct abrasion and exfoliation with adhesive tape (Method II) was unexpected, and may stimulate further research into low-cost methods of resist-free mechanical patterning for linewidths less than state-of-the-art. Abrasion lithography is conceptually and operationally simple and environmentally benign. While abrasion lithography as described in this paper is serial, it could be made parallel by fabricating scoring tools with multiple tips. Using multi-tipped scoring tools, abrasion lithography could become more scalable than is, for example, electrospinning, and could produce better resolution than can proven scalable processes like gravure printing.[63] It also presents advantages when compared to stencil masking because it is impossible to design a stencil mask whose negative areas form a crossbar geometry: the fabrication of grids from stencil masks would require two masking and deposition steps each, whereas abrasion lithography only requires one. Abrasion lithography is in principle compatible with a variety of substrates and methods of deposition. Our initial implementation of abrasion lithography was to pattern by hand, but replacement of the manual processes with automated ones would improve the quality of the structures considerably. Roll-to-roll mechanical patterning of flexible substrates is in principle achievable and is an inviting target of future research. The goal of the project, moreover, was to demonstrate that simple processes and materials could often be used in place of sophisticated tools and specialty chemicals to generate electronic components—*i.e.*, transparent electrodes—whose figures of merit are at least of the same order of magnitude as the state-of-the-art.
